# The relationship between proton pump inhibitors and renal disease

**DOI:** 10.1590/2175-8239-JBN-2018-0021

**Published:** 2018-07-10

**Authors:** Carine Franco Morschel, Denise Mafra, José Carlos Carraro Eduardo

**Affiliations:** 1 Universidade Federal Fluminense, Programa de Pós-Graduação em Ciências Médicas, Niterói, RJ, Brasil.; 2 Universidade Federal Fluminense, Faculdade de Medicina, Niterói, RJ, Brasil.

**Keywords:** Proton Pump Inhibitors, Renal Insufficiency, Chronic, Nephritis, Interstitial, Acute Kidney Injury, Inibidores da Bomba de Prótons, Insuficiência Renal Crônica, Nefrite Intersticial, Lesão Renal Aguda

## Abstract

Proton pump inhibitors (PPIs) bind to enzyme H^+^/K^+^-ATPase and inhibit its activity in the stomach, thus decreasing the secretion of gastric acid. PPIs may trigger acute interstitial nephritis, a potentially severe adverse event commonly associated with acute kidney injury. Studies have found that prolonged use of PPIs may increase the risk of chronic kidney disease (CKD). The increase in prescription and inadequate use of this class of medication calls for studies on the effects of prolonged PPI therapy on renal function. Therefore, this review aimed to analyze recent studies on the matter and discuss the possible consequences of the long-term use of PPIs on renal function.

## RESUMO

Os Inibidores da Bomba de Prótons (IBPs) são medicamentos que inibem a enzima H^+^/K^+^-ATPase no estômago, diminuindo a secreção gástrica. Esses medicamentos podem desencadear nefrite intersticial aguda, evento adverso potencialmente grave e que pode cursar com lesão renal aguda. Além disso, pesquisadores têm observado que o uso prolongado de IBPs pode também aumentar o risco de progressão da doença renal crônica (DRC). Com o crescimento da prescrição e o uso inadequado dessa classe de medicamentos, torna-se importante o estudo dos efeitos do uso prolongado dos IBPs sobre a função renal. Assim, esta revisão pretende abordar os recentes estudos sobre o tema e discutir as possíveis consequências que o uso contínuo dos inibidores da bomba de prótons pode causar na função renal.

Enzyme H^+^/K^+^-ATPase (proton pump), found in the canaliculi of the parietal cells of the stomach, plays a key role in the secretion of hydrochloric acid in the gastric lumen. The enzyme is activated by three distinct stimuli: histamine, gastrin, and acetylcholine. The production of acid occurs with the exchange of K^+^ (potassium) for H^+^ (hydrogen) in an ATP-consuming process.^1^^,^ 2

**Figure 1:**
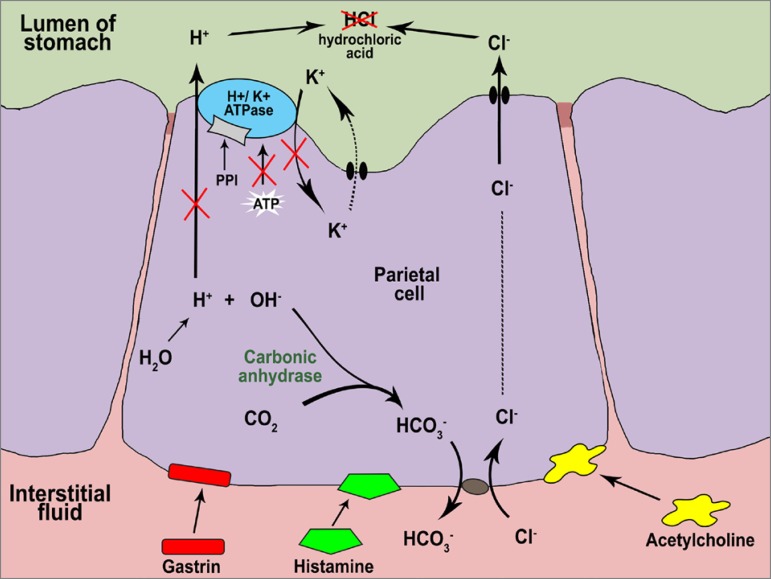
Mode of action of proton pump inhibitors in parietal cells.

PPIs were designed to block acid secretion in the stomach and increase the pH of the gastric juice. They inhibit the action of enzyme H^+^/K^+^-ATPase and prevent the exchange of K^+^ for H^+^, while differentiating themselves from other drugs used to treat gastric diseases for also inhibiting the last step in the production of hydrochloric acid. This process enhances the potency of inhibition, making PPIs the current drug of choice.^2^^-^ 4 PPIs inhibit the enzyme by merging with its receptor and covalently binding to cysteine residues known as irreversible inhibitors (Figure 1). After the reaction, the proton pump cannot regenerate and acid production occurs only after the synthesis of new enzymes. Irreversible inhibition ensures the medication is active for 24 to 48 hours.^3^^,^ 5 ^,^ 6

**Figure 2:**
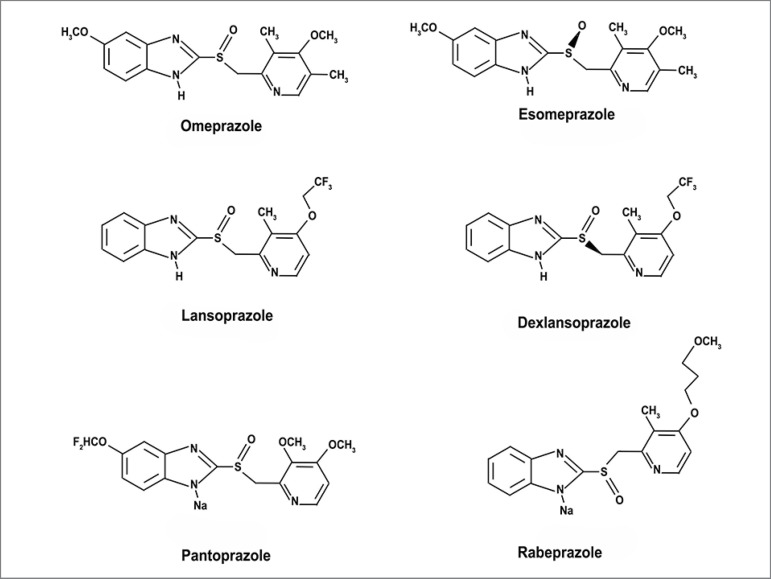
Molecular structures of the proton pump inhibitors available in Brazil.

PPIs are weak acids that share the same basic structure of their molecules, differentiating only in their radicals (Figure 2). They are inactive when administered, and under acidic pH will form sulfamide derivatives or sulfenic acid. PPIs receive gastro-resistant coating to prevent the activation - and thus degradation - of the drug before it arrives at the targeted site. With a plasma half-life of one to two hours, they are quickly absorbed and activated after administration. PPIs are metabolized by liver cytochrome P450 enzymes, which may affect the biotransformation of other medications. In addition, changes in stomach acidity may alter the absorption of other drugs.^1^^-^ 3 ^,^ 7

Omeprazole was the first to be synthesized and is still the most often used drug of this class of medications.^8^ PPIs are prescribed to treat gastric diseases such as gastric and duodenal ulcers, gastroesophageal reflux disease, and erosive esophagitis.^9^

However, as the years went by PPIs began to be prescribed injudiciously to patients outside the scope of indication, for periods longer than recommended, and taken by self-medicating individuals.^4^^,^ 10 In addition, the drug is often used to treat digestive manifestations or to prevent symptoms derived from the use of other medications. All such factors have included PPIs in the list of the most used medications in the world.^11^^,^ 12 ^,^ 13

Side effects are rare. The most common include headache, nausea, constipation, flatulence, diarrhea, skin rash, and dizziness.^4^^,^ 14 Although infrequent, there is growing evidence of other adverse events connected to PPIs such as bone fractures, pneumonia, dementia, hypomagnesemia, and renal diseases including acute interstitial nephritis (AIN), acute kidney injury (AKI), and chronic kidney disease (CKD) more recently.^15^^-^ 17

AIN ranks among the rare adverse events more consistently associated with PPIs. This immune-mediated reaction involves the interstitium and the renal tubules. It may be induced by autoimmune disease, blood disorders, infection, and medication. At first, tubule epithelial cells are injured, and subsequently a lymphocytic inflammatory infiltrate containing predominantly T cells is observed. Renal scarring may initiate as a consequence of the spread of the infiltrate, followed by decrease in renal function. In drug-induced AIN, if no improvement is seen after the discontinuation of the suspected drug and the introduction of corticosteroids, patients may progress to chronic kidney disease with interstitial fibrosis and tubular atrophy.^18^^,^ 19

Although nonspecific symptoms such as malaise, fatigue, weakness, arthralgia, myalgia, fever, and skin rash may occur and confused AIN with other diseases, eosinophilia is a frequent finding. AIN has been estimated to account for eight percent of the cases of acute kidney injury, 70-90% of which induced by medication. The main drug classes linked to AIN are antibiotics, PPIs, and non-steroid anti-inflammatory drugs.^20^^,^ 21

A number of studies published subsequently to the first case study of 1992 supported the link between AIN and use of PPIs.^22^^-^ 24 Antoniou *et al*. reported a three-fold increase in the risk of AIN among individuals taking PPIs (95% CI 1.47-6.14; n = 290,592).^25^ The origin of the renal inflammation seen in these cases has not been established, but the accumulation of PPIs and/or their metabolites in the interstitial tubules and the ensuing immune response have been considered a plausible explanation.^15^

The time until the onset of PPI-induced AIN ranges between hours and months. There is no evident relationship between dosage, latency, time to recovery, age or sex, indicating that this is a condition of immune origin.^26^ Hypersensitivity reaction is apparently a common effect of PPIs, since there are reports of AIN associated with all medications in this drug class.^22^

Differently from AIN induced by other drugs, patients rarely present with the characteristic triad seen in hypersensitivity reaction (fever, skin rash, and eosinophilia). Urinary findings include sterile leukocyturia, hematuria, and urinary eosinophils.^27^ Diagnosis is based on lab and imaging tests and clinical examination, although tests do not necessarily lead to accurate diagnosis.^19^

Treatment to reverse acute disease includes the discontinuation of PPIs, the administration of corticosteroids, and possibly the prescription of renal replacement therapy.21,28 Despite these interventions, over half of the patients cannot fully recover their renal function after AIN. A few are able to return to baseline serum creatinine (Cr) levels, which are slightly increased. Moreover, the estimated glomerular filtration rates of patients recovering from AKI remain below baseline levels.^27^^,^ 29

The fast decrease of renal function derived from tubulointerstitial lesions may promote the onset of AKI. The investigation of the causes of this condition led to the diagnosis of AIN, which is usually confirmed after renal biopsy. When biopsy is contraindicated, patients have the option of undergoing Gallium-67 scintigraphy, a test with great use in the differentiation of AIN from acute tubular necrosis.^30^^,^ 31 Approximately 30% of the patients who recover from AKI remain at increased risk of having CKD.^32^^,^ 33

Hypomagnesemia is another side effect derived from the use of PPIs. A study enrolling 9,818 individuals associated PPIs to a twofold increase in the risk of developing hypomagnesemia (95% CI 1.36-2.93). The mechanism behind the decrease in magnesium (Mg) levels by PPIs has not been entirely elucidated. Low urinary concentrations suggest that Mg depletion occurs in the gastrointestinal tract. Evidence indicates that low blood levels of Mg (< 0.7 mmol/L) are associated with CKD.^20^

**Figure 3:**
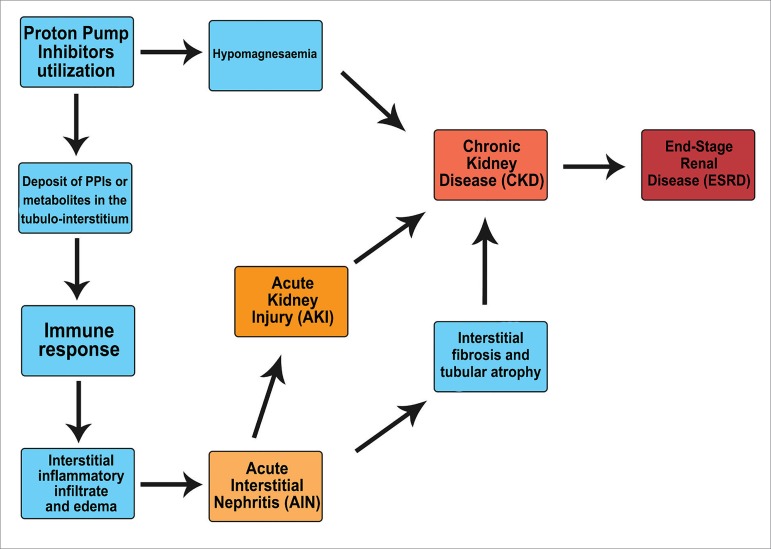
Hypothesis explaining the possible correlation between PPIs and renal disease.

AIN causes acute inflammation and tubulointerstitial damage, which in the long term lead to interstitial fibrosis and chronic interstitial nephritis. Chronic interstitial nephritis may ultimately lead to CKD and, in severe cases, to renal failure. Figure 3 illustrates a hypothesis on how PPIs may cause renal disease.^15^^,^ 20 ^,^ 27

Although few authors have looked into the possible association between PPIs and CKD, the studies published to date have reported increased risk of progression to advanced-stage CKD. A plausible mechanism to explain these findings is still unclear, and authors have considered it a consequence of progression to AIN. The literature indicates that PPIs should be prescribed with caution to patients with CKD, accompanied by creatinine level monitoring where needed.^34^^,^ 17

The first to suggest an association between PPIs and CKD were Lazarus *et al*. in 2016. The authors analyzed whether PPIs alone were and histamine H2-receptor antagonists (H_2_) were not a risk factor for CKD. The authors used the cohort from the Atherosclerosis *Risk in Communities* study with 10,482 participants, and later repeated the study with 248,751 individuals served by the Geisinger Health System. The outcomes were similar in the two groups, and the use of PPIs was associated with increased risk and 1.17 to 1.5 times greater risk of CKD. The association was verified only with PPIs.^35^

Other authors explored the theme in the same year. Peng *et al*. performed a study and analyzed the association between PPIs and risk of progression of renal disease. The study enrolled 7,616 individuals split into two matched groups featuring patients with CKD and controls. The authors found that use of PPIs was more prevalent among patients with CKD, and that the risk of CKD was 1.88 times greater among individuals on PPIs.^34^

Arora *et al*. (2016) conducted a study based on the data of 99,269 individuals from the *New York/ New Jersey Veterans Affairs Health Care* and concluded that patients on PPIs had a greater incidence of CKD and higher mortality risk (OR 1.76; 95% CI 1.67-1.84).^36^

Xie *et al*. published a paper associating PPIs to CKD and progression to renal failure. Participants on PPIs (n = 173,321) or H2RA (n = 20,270) were selected and followed for five years. The study revealed that patients on PPIs were at 1.28 times greater risk of developing CKD and 1.96 times greater risk of progressing to renal failure. As in previous studies, no association was found between H2RA and renal disease.^37^

In 2017, Xie *et al*. published another study based on data from the US Veterans Affairs Department. In this study, 144,032 patients on acid suppression therapy (PPIs and H2RA) were analyzed for AKI renal outcomes without intervention. The occurrence of CKD was 1.26 times greater among patients on PPIs. Similar results were found for declines greater than 30% in the estimated glomerular filtration rate (eGFR) (1.22-time increase) and for advanced-stage CKD or decline of more than 50% in the eGFR (1.30-time increase).^38^

Klatte *et al*. (2017) published a paper with data gathered from the Stockholm CREAtinine Measurements made between 2006 and 2011. In the study design, new users of PPIs (n = 105,305) and H2RA (n = 9,578) were compared for risk of developing CKD. Creatinine levels and CKD progression based on two factors, namely doubling the creatinine level or having a decrease greater than 30% in the eGFR, were analyzed. The results for PPI users revealed risk 1.26 times greater for the two endpoints. Another important finding in this study showed that only 16% of the PPI users had precise indication for treatment.^39^

It is important to note that all publications looking into the matter are observational studies based on retrospective data, thus subject to significant bias. To this date, randomized clinical trials have not been performed to shed more light on the issue. With that in mind, Tomlinson *et al*. (2017) stressed that despite the correlations described between the use of PPIs and CKD progression, the evidence to support this causal relationship is not strong. The authors suggested that more detailed studies are required to confirm the increase in risk. They added that patients have been taking PPIs for a long time without specific treatment indication and that the cost-effectiveness of these medications should be assessed.^40^

The associations between AIN, AKI, and PPIs, although more significant in the literature than with CKD are, likewise, the topic of retrospective studies, from which a causal relationship cannot be extracted.^18^^,^ 25 ^,^ 41 However, the correlation between PPIs and renal disease is plausible. Randomized clinical trials are needed to consolidate the possible effects of this drug class on renal function.

Several studies have alluded to the inadequate administration of PPIs, often the object of off-label therapies, unnecessarily long courses of treatment, and self-medication.^13^^,^ 42 ^-^ 44 Although patients in Brazil are required to produce a medical prescription to buy PPIs, drugs in this class can be unreservedly acquired in drugstores all over the nation.^15^^,^ 16 ^,^ 45 ^,^ 46 In this context, healthcare workers and the general population must be made aware of the risks associated with the use of medication without professional advice and its ensuing impacts on public health.^47^

The medical relevance, along with the efficacy and safety of PPIs, cannot be denied. Proper use of the medication must be enforced in accordance with therapeutic guidelines. The benefits yielded by PPIs must be monitored, and drug therapy discontinued as soon as it is no longer needed.^15^^,^ 48 In clinical practice, Nehra, Alexander, and Loftus suggested that the eGFR be monitored annually, as recommended for patients on potentially nephrotoxic drugs.^49^

## References

[1] Shin JM, Sachs G (2008). Pharmacology of proton pump inhibitors. Curr Gastroenterol Rep.

[2] Roche VF (2006). The chemically elegant proton pump inhibitors. Am J Pharm Educ.

[3] Strand DS, Kim D, Peura DA (2017). 25 Years of Proton Pump Inhibitors: A Comprehensive
Review. Gut Liver.

[4] Braga MP, da Silva C de B, Adams AIH (2011). Inibidores da bomba de prótons: revisão e análise
farmacoeconômica. Saúde.

[5] Brinkworth MD, Aouthmany M, Sheehan M (2016). Histamine 2 Receptor Antagonists and Proton Pump
Inhibitors. Dermatitis.

[6] Shin JM, Kim N (2013). Pharmacokinetics and pharmacodynamics of the proton pump
inhibitors. J Neurogastroenterol Motil.

[7] Câmara de Regulação do Mercado de Medicamentos C (2017). Preços máximos de medicamentos por princípio ativo.

[8] Brewster UC, Perazella MA (2007). Acute kidney injury following proton pump inhibitor
therapy. Kidney Int.

[9] Nadri Q, Althaf MM (2014). Granulomatous tubulointerstitial nephritis secondary to
omeprazole. BMJ Case Rep.

[10] George CJ, Korc B, Ross JS (2008). Appropriate Proton Pump Inhibitor Use Among Older Adults: A
Retrospective Chart Review. Am J Geriatr Pharmacother.

[11] Yadlapati R, Kahrilas PJ (2017). When is proton pump inhibitor use appropriate?. BMC Med.

[12] Wannmacher L (2004). Inibidores da bomba de prótons: Indicações
racionais. Uso Racion Medicam Temas Selecionados.

[13] Scarpignato C, Gatta L, Zullo A, Blandizzi C, SIF-AIGO-FIMMG Group, Italian Society of Pharmacology, the Italian Association of Hospital
Gastroenterologists, and the Italian Federation of General
Practitioners (2016). Effective and safe proton pump inhibitor therapy in acid-related
diseases - A position paper addressing benefits and potential harms of acid
suppression. BMC Med.

[14] Aronson JK (2016). Inhibiting the proton pump: mechanisms, benefits, harms, and
questions. BMC Med.

[15] Eusebi LH, Rabitti S, Artesiani ML, Gelli D, Montagnani M, Zagari RM (2017). Proton pump inhibitors: Risks of long-term use. J Gastroenterol Hepatol.

[16] Nochaiwong S, Ruengorn C, Awiphan R, Koyratkoson K, Chaisai C, Noppakun K (2018). The association between proton pump inhibitor use and the risk of
adverse kidney outcomes: a systematic review and
meta-analysis. Nephrol Dial Transplant.

[17] Schnoll-Sussman F, Katz PO (2017). Clinical Implications of Emerging Data on the Safety of Proton
Pump Inhibitors. Curr Treat Options Gastroenterol.

[18] Nast CC (2017). Medication-Induced Interstitial Nephritis in the 21st
Century. Adv Chronic Kidney Dis.

[19] Perazella MA (2017). Clinical Approach to Diagnosing Acute and Chronic
Tubulointerstitial Disease. Adv Chronic Kidney Dis.

[20] Malavade P, Hiremath S (2017). Proton pump inhibitors: More Indigestion than
Relief?. Indian J Nephrol.

[21] Ramachandran R, Kumar K, Nada R, Jha V, Gupta K, Kohli H (2015). Drug-induced acute interstitial nephritis: A clinicopathological
study and comparative trial of steroid regimens. Indian J Nephrol.

[22] Sampathkumar K, Ramalingam R, Prabakar A, Abraham A (2013). Acute interstitial nephritis due to proton pump
inhibitors. Indian J Nephrol.

[23] Torregrosa E, Rovira R, Calvo C, Hernández-Jaras J, Maduell F, García H (2004). Nefritis intersticial aguda por omeprazol. Nefrología.

[24] Simpson IJ, Marshall MR, Pilmore H, Manley P, Williams L, Thein H (2006). Proton pump inhibitors and acute interstitial nephritis: report
and analysis of 15 cases. Nephrology.

[25] Antoniou T, Macdonald EM, Hollands S, Gomes T, Mamdani MM, Garg AX (2015). Proton pump inhibitors and the risk of acute kidney injury in
older patients: a population-based cohort study. CMAJ Open.

[26] Raghavan R, Shawar S (2017). Mechanisms of Drug-Induced Interstitial Nephritis. Adv Chronic Kidney Dis.

[27] Moledina DG, Perazella MA (2016). PPIs and kidney disease: from AIN to CKD. J Nephrol.

[28] Härmark L, van Der Wiel HE, de Groot MCH, van Grootheest AC (2007). Proton pump inhibitor-induced acute interstitial
nephritis. Br J Clin Pharmacol.

[29] Torpey N, Barker T, Ross C (2004). Drug-induced tubulo-interstitial nephritis secondary to proton
pump inhibitors: experience from a single UK renal unit. Nephrol Dial Transplant.

[30] Graham F, Lord M, Froment D, Cardinal H, Bollée G (2016). The use of gallium-67 scintigraphy in the diagnosis of acute
interstitial nephritis. Clin Kidney J.

[31] Kodner CM, Kudrimoti A (2003). Diagnosis and management of acute interstitial
nephritis. Am Fam Physician.

[32] KDIGO (2012). KDIGO Clinical Practice Guideline for Acute Kidney
Injury. Kidney Int Suppl.

[33] Praga M, González E (2010). Acute interstitial nephritis. Kidney Int.

[34] Peng YC, Lin CL, Yeh HZ, Chang CS, Wu YL, Kao CH (2016). Association Between the Use of Proton Pump Inhibitors and the
Risk of ESRD in Renal Diseases: A Population-Based, Case-Control
Study. Medicine.

[35] Lazarus B, Chen Y, Wilson FP, Sang Y, Chang AR, Coresh J (2016). Proton Pump Inhibitor Use and the Risk of Chronic Kidney
Disease. JAMA Intern Med.

[36] Arora P, Gupta A, Golzy M, Patel N, Carter RL, Jalal K (2016). Proton pump inhibitors are associated with increased risk of
development of chronic kidney disease. BMC Nephrol.

[37] Xie Y, Bowe B, Li T, Xian H, Balasubramanian S, Al-Aly Z (2016). Proton Pump Inhibitors and Risk of Incident CKD and Progression
to ESRD. J Am Soc Nephrol.

[38] Xie Y, Bowe B, Li T, Xian H, Yan Y, Al-Aly Z (2017). Long-term kidney outcomes among users of proton pump inhibitors
without intervening acute kidney injury. Kidney Int.

[39] Klatte DCF, Gasparini A, Xu H, de Deco P, Trevisan M, Johansson ALV (2017). Association Between Proton Pump Inhibitor Use and Risk of
Progression of Chronic Kidney Disease. Gastroenterology.

[40] Tomlinson LA, Fogarty DG, Douglas I, Nitsch D (2017). Pharmacoepidemiology for nephrologists: do proton pump inhibitors
cause chronic kidney disease?. Nephrol Dial Transplant.

[41] Muriithi AK, Leung N, Valeri AM, Cornell LD, Sethi S, Fidler ME (2014). Biopsy-proven acute interstitial nephritis, 1993-2011: A case
series. Am J Kidney Dis.

[42] Braga DC, Bortolini SM, Stroher CK, Cassol M, Bordignon S, Byczkovski T (2014). Uso crônico de inibidores da bomba de prótons na atenção
primária. GED Gastroenterol Endosc Dig.

[43] Garcia M, Saracho R, Jaio N, Vrotsoukanari K, Aguirre C (2010). Inadequate drug prescription and the rise in drug-induced acute
tubulointerstitial nephritis incidence. NDT Plus.

[44] Divisón Garrote JA, Escobar Cervantes C (2017). Uso de inhibidor de la bomba de protones y el riesgo de
enfermedad renal crónica. Semergen.

[45] Yang Y, George KC, Shang WF, Zeng R, Ge SW, Xu G (2017). Proton-pump inhibitors use, and risk of acute kidney injury: a
meta-analysis of observational studies. Drug Des Devel Ther.

[46] Brasil, Ministério da Saúde, Anvisa (2016). Instrução Normativa N.11 - Lista de medicamentos isentos de
prescrição.

[47] Pereira JR, Soares L, Hoepfner L, Kruger KE, Guttervil ML, Tonini KC (2008). Riscos da Automedicação: Tratando o problema com conhecimento.

[48] Toth-Manikowski S, Grams ME (2017). Proton Pump Inhibitors and Kidney Disease-GI Upset for the
Nephrologist?. Kidney Int Rep.

[49] Nehra AK, Alexander JA, Loftus CG, Nehra V (2018). Proton Pump Inhibitors: Review of Emerging
Concerns. Mayo Clin Proc.

